# When preparation pays off

**DOI:** 10.7554/eLife.102187

**Published:** 2024-09-23

**Authors:** Mark M Churchland

**Affiliations:** 1 Grossman Center for the Statistics of Mind, Columbia University in the City of New York New York United States; 2 Kavli Institute for Brain Science, Columbia University in the City of New York New York United States; 3 Department of Neuroscience, Columbia University in the City of New York New York United States

**Keywords:** recurrent neural networks, motor control, motor preparation, optimal control, delayed reaching, motor networks, None

## Abstract

Computational principles shed light on why movement is preceded by preparatory activity within the neural networks that control muscles.

**Related research article** Schimel M, Kao CT, Hennequin G. 2023. When and why does motor preparation arise in recurrent neural network models of motor control?. *eLife* **12**:RP89131. doi: 10.7554/eLife.89131.

When we indulge in a mid-afternoon snack, neurons in our motor and premotor cortex are active well before we extend our arm to obtain the desired treat (Tanji and Evarts, 1976; Churchland and Shenoy, 2024). Multiple lines of evidence suggest that this ‘preparatory activity’ is the first stage in a chain of events that generates movement (Churchland and Shenoy, 2024; Sussillo et al., 2015; Inagaki et al., 2022). According to this model, descending inputs from higher cognitive areas arrive early, generating preparatory activity in the neural networks that control muscles. A triggering input then changes the dynamics of the network, prompting execution of the prepared movement.

Much like the initial position and velocity of an object determine its future trajectory, preparatory activity is thought to ‘seed’ the neural trajectory during execution. That trajectory is proposed to generate movement-producing commands (Churchland et al., 2012; Russo et al., 2018) and to determine the network’s control policy – how commands are adjusted based on sensory feedback during movement (Saxena et al., 2022; Pruszynski and Scott, 2012). Taken to the extreme, the movement and its control policy could be fully determined by the state of the network just before execution begins, with no additional descending inputs required during action. Indeed, neural activity in ‘preparatory dimensions’ (which are presumed to reflect descending inputs) becomes diminished during successful movement, only increasing if corrections or additional actions are needed (Ames et al., 2019; Stavisky et al., 2017; Zimnik and Churchland, 2021).

Because preparatory activity is consistently present before voluntary movement (Lara et al., 2018), it is tempting to think that nature had to use this strategy. Yet motor networks could, in principle, use a range of approaches. Movement could be specified by descending inputs that occur solely during preparation, or solely during execution. Given these options, why would nature choose the preparatory strategy, especially when the time used to prepare might sometimes be the difference between success and failure? Modeling approaches can help answer these questions by allowing scientists to build networks that use a range of strategies, then assess which one is ‘best’. Now, in eLife, Marine Schimel (University of Cambridge), Ta-Chu Kao (Meta Reality Labs) and Guillaume Hennequin (University of Cambridge) report the results of modeling work that allowed them to explore why nature prepares (Schimel et al., 2023).

The team built networks that simulate the dynamics of the motor cortex and generate muscle commands to control an arm as it reaches towards one of eight targets. Networks rely on descending inputs specific to the desired reach target. Schimel et al. allow such inputs to arrive at any time before or during the reach. This creates a spectrum of possibilities, from movement being entirely determined by preparation (no input required during action) to completely unprepared (no inputs arriving early). To assess which is best, Schimel et al. make reasonable assumptions about what nature is trying to do and what it is trying to avoid: in formal terms, they create a ‘cost function’. Cost is increased if the movement takes too long, is premature, or is inaccurate. Another assumption is that descending inputs to the motor cortex should be small and/or simple. This makes sense: if descending inputs had no limitations, there would be no need for a motor cortex. To formalize this intuition, cost rises if inputs are too large or change too swiftly. The central finding of the study is that the total cost is smallest when inputs arrive early, creating a preparatory stage. These early inputs created the best balance between needing to be accurate, needing to be fast, and needing to use small and/or simple descending inputs. Thus, the strategy of preparing is optimal, likely explaining why nature uses it.

Schimel et al. establish that two basic properties of recurrent networks cause preparation to be a good strategy. First, descending inputs must be able to alter network activity without producing an immediate change in output. In technical terms, there must exist ‘output-null dimensions’, so that preparation can exist without causing movement. Networks must also have strong dynamics, such that preparatory activity can shape future outputs. Because such dynamics are deeply intertwined with the benefits of preparation, the team was able to predict the strength of preparatory activity for a given network based on features of its dynamics. Preparation is pronounced when output-null dimensions strongly influence future network output, making preparation effective. Additionally, preparation is pronounced when options for controlling network output (via descending inputs) are generally limited, making non-preparation-based strategies less effective.

Until the present study, network models have not attempted to explain why preparation exists – they have simply assumed that it does. Yet the preparation-encouraging properties highlighted by Schimel et al. occur in many current models (Hennequin et al., 2014; Sussillo et al., 2015; Saxena et al., 2022). The finding that preparation is optimal therefore justifies, in retrospect, the choice to supply networks with distinct preparatory inputs during a dedicated preparatory stage. Also based on principles of optimality, the study is able to explain qualitative features of preparation during movement sequences (a series of movements performed in a given order). Although one might have expected a single bout of preparation before a swift two-reach sequence, empirically each reach is preceded by its own dedicated preparatory phase. This strategy is naturally reproduced by the simulated networks.

Overall, the work by Schimel et al. reshapes our understanding of preparation. Historically, this process has been considered a distinct stage where movement is programmed. The new proposed framework absorbs aspects of this view, yet suggests that preparation is a special case of a broader phenomenon. Given many output-null dimensions and strong dynamics (Churchland and Shenoy, 2024; Kaufman et al., 2014), inputs can alter motor cortex activity in ways that do not immediately alter network output yet could facilitate future output. This strategy may be used not only during preparation but also during corrections, when canceling movement, and when stringing multiple movements together (Ames et al., 2019; Stavisky et al., 2017; Zimnik and Churchland, 2021; Pani et al., 2022).
